# Coffee break has no impact on laparoscopic skills: a randomized double-blinded placebo-controlled parallel-group trial

**DOI:** 10.1007/s00464-021-08675-9

**Published:** 2021-08-30

**Authors:** Christoph Gerdes, Anna Maria Berghäuser, Julian Hipp, Martin Bäumlein, Svenja Hinrichs, Jan-Christoph Thomassen, Sebastian Hoffmann, Berthold Gerdes

**Affiliations:** 1 Organization Team of the Skills Lab of the German Society of Surgery (DGCH), Berlin, Germany; 2 Department of General Surgery, Philipps University Marburg, Marburg, Germany; 3 Department of General Surgery, Johannes Wesling University Hospital, Hans-Nolte-Str. 1, 32429 Minden, Germany

**Keywords:** Coffee, Caffeine, Surgery, Laparoscopy, Tremor, Fine motor skills

## Abstract

**Background:**

Coffee is a widely consumed beverage. Surgeons often drink coffee before performing surgery. Caffeine intake leads to tremor which might have a negative effect on surgeons’ fine motor skills.

**Methods:**

A double-blinded parallel-group trial was conducted in order to investigate if caffeinated coffee intake has a negative effect on laparoscopic skills and increases tremor, regardless of previous coffee consumption. 118 participants were selected during a congress of the German Society of Surgery. Exclusion criteria were immaturity and no given consent. Participants and investigators were blinded. Participants were randomized with a 1:1 allocation into interventional group receiving caffeinated coffee or placebo group receiving decaffeinated coffee. The motor skills were tested with two validated laparoscopic exercises at a laparoscopy simulator (LapSim®) before and 30 min after coffee intake. Data on influencing factors were recorded in a standardized questionnaire and tested for equal distribution in both groups. In both exercises four parameters were recorded: left and right hand path length and angular path. Their differences and the resulting effect scores were calculated for both groups as primary outcome to test which group showed greater improvement on the second round of exercises. Registration number DRKS00023608, registered retrospectively.

**Results:**

Fifty nine subjects were assigned to each the interventional (54 analyzed) and placebo group (53 analyzed) with 11 drop outs. There was no significant difference between the placebo and interventional group in the two exercises in effect score 30 min after coffee intake [mean (SD); 38.58 (10.66) vs. 41.73 (7.40) and 113.09 (28.94) vs. 116.59 (25.63)]. A significant improvement from first to second measurement in the first exercise could be observed for both groups, demonstrating the training effect.

**Conclusion:**

In our study, we verified that additional caffeinated coffee intake, e.g., during a coffee break, does not lead to deterioration of laparoscopic fine motor skills.

**Supplementary Information:**

The online version contains supplementary material available at 10.1007/s00464-021-08675-9.

Coffee is a popular beverage in adults for a range of reasons, including taste or cognitive enhancement. A cup of 125 ml coffee contains about 83–125 mg caffeine [1, 2]. Oral intake of caffeine leads to a fast increase of serum caffeine levels, reaching a maximum concentration after about 30 min. The bioavailability is nearly 100% [1, 3]. Degradation is more variable, with biological half-lives between 2.7 and 9.9 h. In plasma, caffeine binds to albumine [4]. In the liver, it is metabolized mainly to paraxanthin, theobromin, and theophylline [5]. The extent of metabolism varies, depending on CYP1A2-variability, which can be either caused by genetic or environmental factors, such as smoking, alcohol, contraceptives, and gravidity [6]. The main pharmacological effects of caffeine are due to a blockage of adenosine-receptors [5].

A recent study demonstrated that more than half of the surgeons consumed coffee in the last week, mostly in order to cope with fatigue [7], and surgeons have the highest coffee consumption of all doctors. We sought to investigate whether caffeine might influence surgical skills. An interventional study on 18 students demonstrated that in sleep-deprived participants caffeine led to improvement of reaction time and overall time taken and may restore psychomotor functions to rested levels, however, it did not lead to a reduction in error rate [8]. In various studies, caffeine was found to improve psychomotor performance and cognitive skills [9–11].

However, coffee might not solely prove beneficial or irrelevant for performing surgery, instead having a negative impact on surgical skills. Consumption of more than four cups of coffee, i.e., about 500–600 mg caffeine, may lead to sleeplessness, nervousness, restlessness, tremor, indigestion, and tachycardia [12]. Of these, tremor is the symptom which might be the most relevant in impairing surgical skills. 2% of people notice a tremor after drinking coffee [13] and caffeine consumption increases whole-arm-tremor [14]. Tremor as a consequence of coffee consumption and its effect on surgery performance have not been widely studied. Studies have been performed especially in ophthalmologists and while some studies found that surgeon hand tremor is not influenced by caffeine [15–17], others advise against the use of coffee before surgery [18, 19].

In this study, we explore the effect of coffee consumption on laparoscopic surgical skills. Participants were required to consume either caffeinated or decaffeinated coffee after a round of virtual laparoscopic exercises, and our objective was to test which group showed greater improvement on the second round of exercises.

## Methods

### Trial design and intervention

**Fig. 1:**
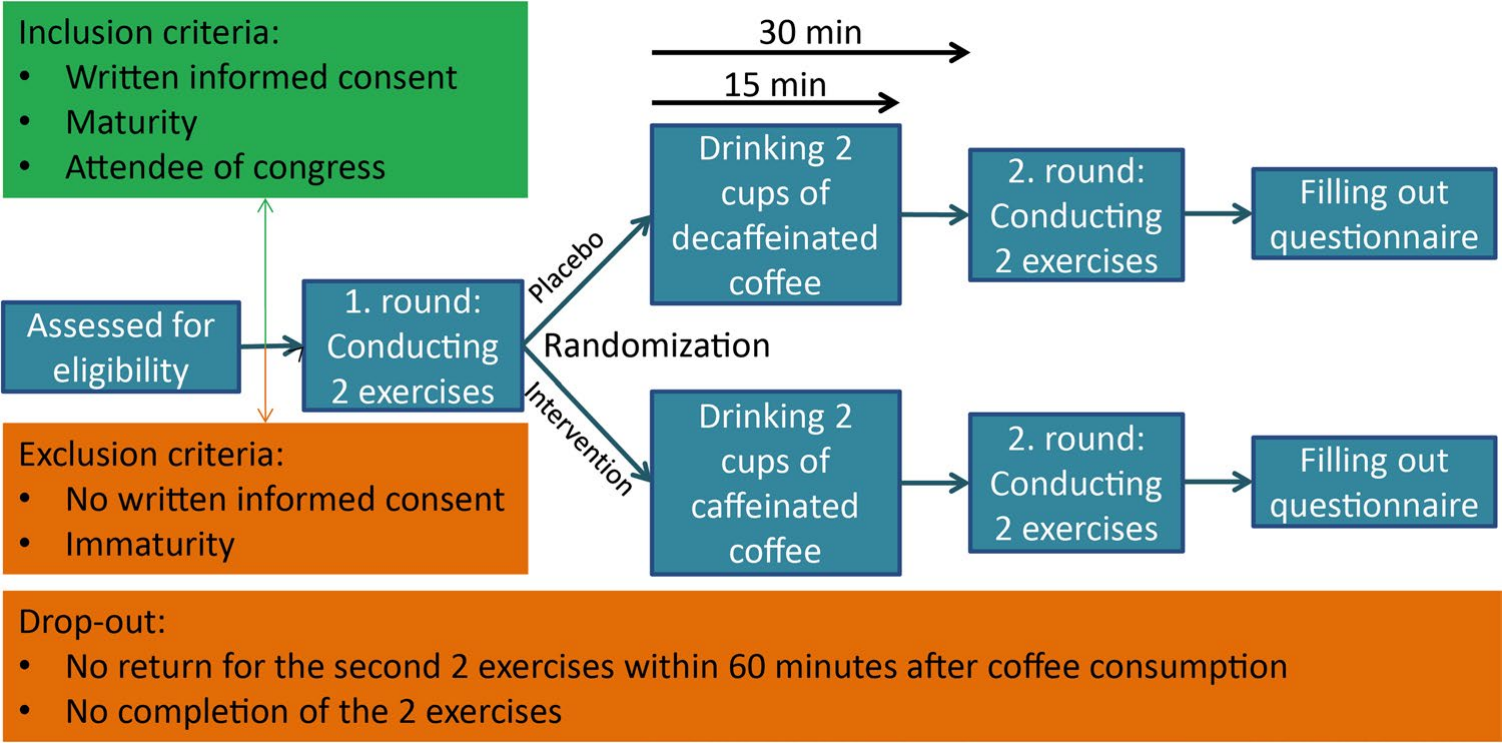
Flowchart of the trial design. After conducting the first round of performing exercises, the subjects were randomized into control (upper boxes) and interventional group (lower boxes). Two cups of coffee were defined as 340 ml

The study was conducted as a randomized double-blinded placebo-controlled parallel-group study (Fig. 1) with the interventional arm receiving caffeinated coffee and the placebo arm receiving decaffeinated coffee.

To measure motoric skills, we used laparoscopic simulators. Surgical simulators provide a possibility to measure surgical skills objectively and improve surgical skills [20, 21]. In our study, the simulator LapSim® (Surgical Science, Gothenburg, Sweden) was used. This simulator has been validated in several studies for content validity [22], concurrent validity [23], construct validity [24–26], and face validity [24]. The two exercises which had to be conducted by each participant were ‘Lifting and Grasping’ and ‘Clip Applying’.

Following written informed consent, the participants conducted a first round of two exercises at the LapSim®, namely ‘Lifting and Grasping’ and ‘Clip applying’. In order to achieve comparable study groups, participants were then assigned to one of two groups with a 1:1 allocation by simple randomization generated by the investigators. Investigators enrolled participants and assigned them to interventions. Control group was given decaffeinated coffee and interventional group caffeinated coffee (Tchibo GmbH, Hamburg, Germany) prepared with a conventional coffee machine. Both, participants and investigators were blinded. Groups were marked as A or B. Participants consumed two cups of coffee (340 ml) in 15 min. 30 min after starting consuming the coffee, the exercises were conducted again (second round). Data on influencing factors such as age, gender, laparoscopy experience, smoking, coffee intake before starting the study, were recorded in a standardized questionnaire and tested for equal distribution in both, interventional and control groups.

### Participants and study setting

One hundred and eighteen participants were recruited during the four days of the annual congress of the German Society of Surgery (Deutsche Gesellschaft für Chirurgie, Berlin). Any attendee of the congress could participate in the study. Exclusion criteria were immaturity (age < 18 years, incapability to give consent) and no given consent. Participants were enrolled given their ability to understand the extent and nature of the trial, and their written informed consent after detailed participant information. This study was conducted in agreement with the Declaration of Helsinki in its current version and was approved by the ethical committee of the Philipps-University Marburg.

### Outcomes

The primary endpoint was the difference of the calculated effect score between the two groups, representing the difference in improvement from first to second measurement depending on caffeine consumption. For measuring manual dexterity and thereby fine motor skills, left and right hand path length (LIPL, RIPL) and angular path (LIAP, RIAP) are validated variables and have therefore been measured [27, 28]. For calculation of a total effect score the differences of LIPL, RIPL, LIAP, and RIAP between first and second round were calculated. These differences were classified for path length in 0.1 m steps and for angular path in 25° angles. After classification these were added to a total effect score: (classified LIPL difference + classified RIPL difference + classified LIAP difference + classified RIAP difference) × 0.25. The smaller the effect score, the smaller the difference between first and second round. This was done in order to calculate an overall score by including all single parameters.

We also performed a subgroup analysis of participants who had abstained from coffee for at least 8 h before commencement of the study, and a subgroup analysis of experienced surgeons (> 100 laparoscopies). Secondary endpoint was improvement in single hand parameters from first to second round of exercises.

### Sample size calculation

Post hoc sample size calculation revealed, that the recruited sample size was adequate to demonstrate non-inferiority of the interventional group compared to the control group at a non-inferiority margin of 13.3% (‘Lifting and Grasping’) and 12.3% (‘Clip Applying’) of the total effect score at a significance level of 5% with a power of 80% [29].

### Statistical analysis

Continuous data were checked for normal distribution by the Shapiro–Wilk test and compared using the unpaired two-sided *t* test or paired two-sided *t* test, as applicable, and categorical data with the chi-squared test. *p* < 0.05 was considered statistically significant. Statistical analyses were performed using IBM SPSS Statistics Version 20 (Armonk, NY, USA) and GraphPad Prism 5 (San Diego, CA, USA). All numbers are given as mean ± standard deviation, unless otherwise specified.

## Results

### Characteristics of participants and trial design validation

**Fig. 2:**
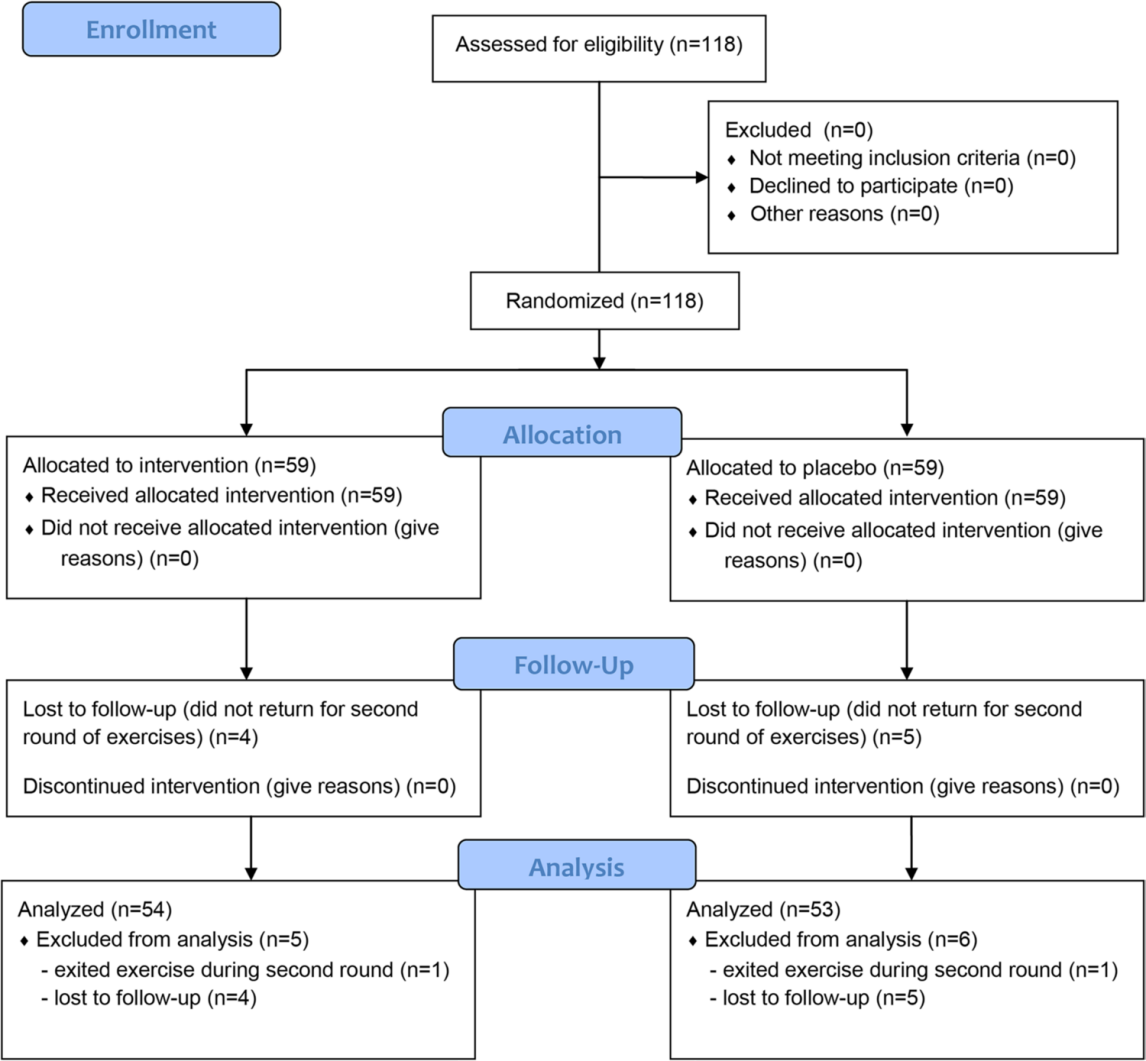
CONSORT 2010 flow diagram of the trial

One hundred and eighteen study participants were enrolled in the study of which 11 dropped out due to lost to follow-up or not completing the exercises. Thus, 107 subjects were analyzed, of which 53 (49.5%) were assigned to the interventional group and 54 (50.5%) to the control group (Fig. 2).

In the first round of exercises before drinking coffee, unpaired two-sided *t* test showed no significant difference between the two groups in means of each single measurement, neither in ‘Lifting and Grasping’ [LIPL (*p* = 0.159), LIAP (*p* = 0.172), RIPL (*p* = 0.234), RIAP (*p* = 0.161)] nor in ‘Clip Applying’ [LIPL (*p* = 0.127), LIAP (*p* = 0.195), RIPL (*p* = 0.360), RIAP (*p* = 0.279)]. Therefore, both groups had a similar base level in surgical skills.

Characteristics of participants are shown in Table 1. Unpaired two-sided *t* test or chi-squared test revealed no significant differences and that these were equally distributed. Thus, the two arms only differed in the additional consumption of caffeinated vs. decaffeinated coffee.

**Table 1:** Participant characteristics

	Decaffeinated (control) *n* = 54	Caffeinated (interventional) *n* = 53	All *n* = 107	*p* value
Age [years]	*n* = 50	*n* = 49	*n* = 99	0.992*
	33.08 (9.06)	33.06 (9.05)	33.07 (9.01)	
Gender	*n* = 53	*n* = 53	*n* = 106	0.842#
Male	32 (60.4%)	33 (62.3%)	65 (61.3%)	
Female	21 (39.6%)	20 (37.7%)	41 (38.7%)	
Physician	33 (61.1%)	36 (67.9%)	69 (64.5%)	0.462#
Laparoscopic experience				0.523#
< 10 laparoscopies	27 (50%)	25 (47.2%)	52 (48.6%)	
10–50	11(20.4%)	8 (15.1%)	19 (17.8%)	
51–100	6 (11.1%)	7 (13.2%)	13 (12.1%)	
> 100	10 (18.5%)	13 (24.5%)	23 (21.5%)	
Smoker	*n* = 53	*n* = 50	*n* = 103	0.419#
	13 (24.5%)	9 (18.0%)	22 (21.4%)	
Coffee per day [cups]	*n* = 52	*n* = 52	*n* = 104	0.524*
	3.20 (3.18)	2.85 (2.45)	3.02 (2.83)	
Coffee at study day [cups]	*n* = 51	*n* = 51	*n* = 102	0.704*
	1.49 (1.08)	1.41 (1.49)	1.45 (1.29)	
Last coffee [hours before study in the last 24 h]	*n* = 52	*n* = 51	*n* = 103	0.264*
	5.74 (11.03)	3.57 (5.15)	4.81 (9.03)	
Opinion whether caffeine influences motoric skills	*n* = 53	*n* = 51	*n* = 104	0.283#
Yes	31 (58.5%)	35 (68.6%)	66 (63.5%)	
No	22 (41.5%)	16 (31.4%)	38 (36.5%)	

No significant differences were detected between control and interventional group in all categories using two-sided unpaired *t* test for continuous data and chi-squared test for categorical data. Numbers in parentheses represent standard deviation unless otherwise stated

*Chi-squared test

^#^Two-sided unpaired *t* test

To control whether our blinding and placebo worked, we asked participants if—in their opinion—they had drunk caffeinated or decaffeinated coffee. There was no significant difference between the two groups, thus revealing that the blinding had worked sufficiently (*p* = 0.073). Furthermore, we controlled whether participants began the second round of exercises 30 min after they had begun drinking coffee, as detailed in the trial design, and found that they started 32 ± 4 min after begin of coffee consumption; therefore, being in a reasonable time frame.

### Primary endpoint: no influence on manual dexterity by caffeinated coffee

**Fig. 3:**
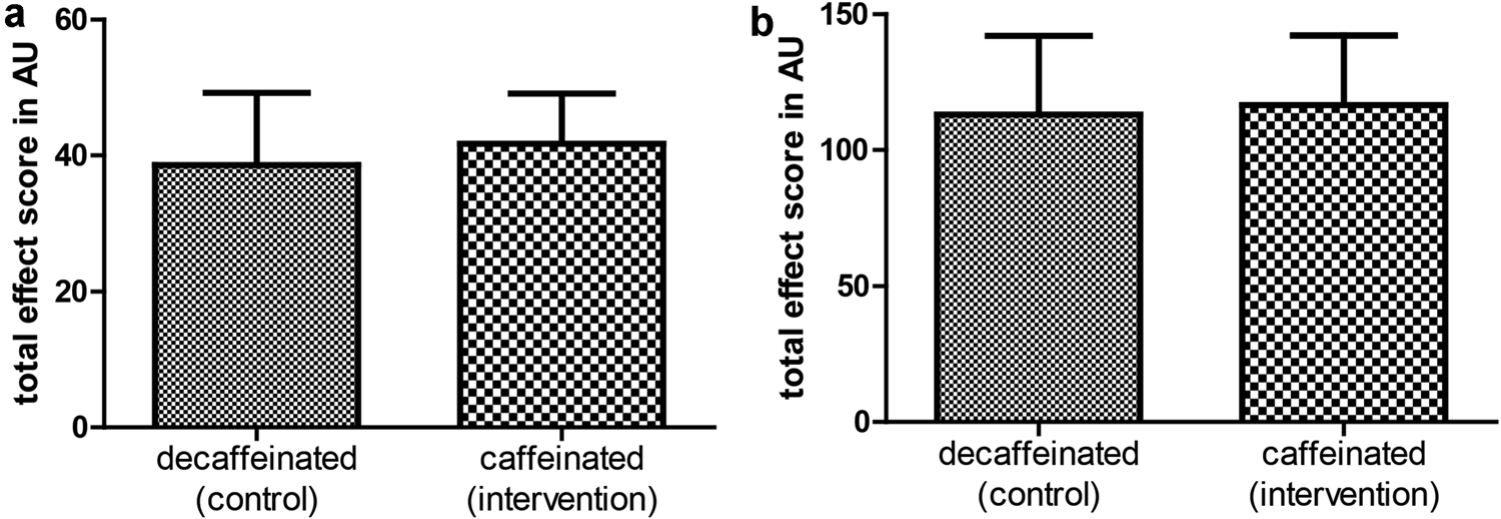
No influence of caffeinated coffee on laparoscopic hand skills. Statistical analysis was performed using unpaired two-sided *t* test. Total effect score is the combined classified difference in path length and angular path of each participant in arbitrary units (AU). **a** No significant difference in hand movement for ‚Lifting and Grasping ‘ [intervention 41.73 (7.40) vs. control 38.58 (10.66); *p* = 0.079]. **b** No significant difference in hand movement for ‚Clip Applying ‘ [intervention 116.59 (25.63) vs. control 113.09 (28.94); *p* = 0.511]

Having excluded other confounders and verified correct conduction of our trial design, we tested for differences in improvement of surgical skills between control and interventional group after they had drunk decaffeinated or caffeinated coffee, respectively. Our analysis revealed no significant difference between the two groups in effect score. Therefore, non-inferiority of laparoscopic skills after consumption of caffeinated coffee compared to consumption of decaffeinated coffee could be demonstrated (Fig. 3).

We also made an exploratory subgroup analysis if caffeinated coffee had an effect on the 25 participants, who had abstained from caffeine for at least 8 h as caffeine should be mainly degraded by then and this cut-off was used in another study as well [18]. Of these, 9 had received the placebo and 16 the caffeinated coffee. It revealed that there was no significant difference in effect score between placebo and interventional group for ‘Lifting and Grasping’ [39.42 (9.10) vs. 39.28 (6.81); *p* = 0.967] as well as ‘Clip Applying’ [110.30 (15.22) vs. 110.30 (22.89); *p* = 0.999].

To evaluate if the effect of caffeine was different in the 23 participants, who had extensive previous laparoscopic experience, we made another exploratory subgroup analysis. We compared participants with high laparoscopic experience (> 100 laparoscopies) who either did (*n* = 10) or did not (*n* = 13) drink caffeinated coffee. The mean age of this group was higher than the total mean age [42.11 (6.76) vs. 33.07 (9.01) years]. We found that there was no significant difference in ‘Lifting and Grasping’, though there was a slightly higher effect score and thus less tremor in the interventional group [38.15 (2.86) vs. 43.87 (1.43); *p* = 0.068]. There was no significant difference in effect score between control and interventional group in ‘Clip Applying’ [120.20 (10.70) vs. 117.30 (3.03); *p* = 0.771].

### Secondary endpoint: simulator shows training effect

**Fig. 4:**
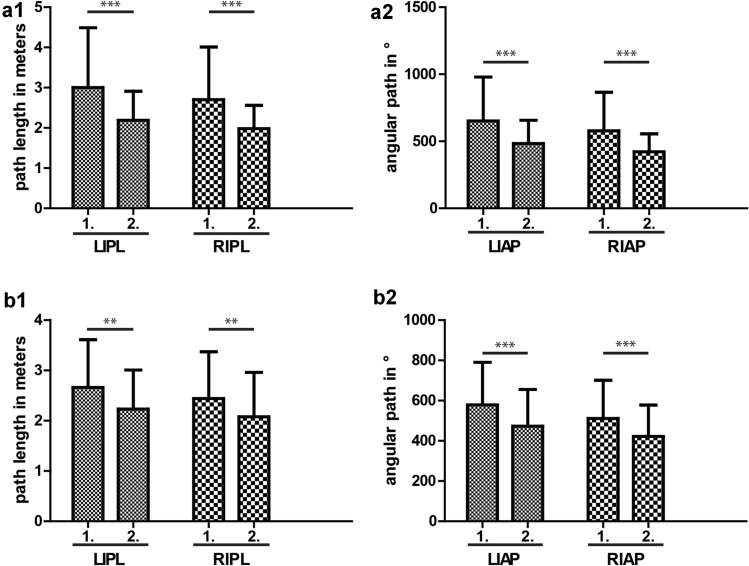
Comparison of first to second round of ‘Lifting and Grasping’ exercise. Shorter path length and smaller angular path mean more manual dexterity. Statistical analysis was made using two-sided paired *t* testing, **p < 0.01, ***p < 0.001. a—Placebo group. **a1** Path length. Subjects improved significantly from first to second round in path length [LIPL 3.00 (1.49) vs. 2.19 (0.72), *p* < 0.001; RIPL 2.70 (1.31) vs. 1.98 (0.58), *p* < 0.001]. **a2** Angular path. Subjects improved significantly from first to second round in angular path [LIAP 652.40 (327.33) vs. 483.01 (173.34), *p* < 0.001; RIAP 577.95 (287.91) vs. 421.63 (134.09), *p* < 0.001]. b—Interventional group. **b1** Path length. Subjects improved significantly from first to second round in path length [LIPL 2.66 (0.95) vs. 2.23 (0.78), *p* = 0.001; RIPL 2.44 (± 0.93) vs. 2.08 (0.88), *p* = 0.003]. **b2** Angular path. Subjects improved significantly from first to second round in angular path [LIAP 579.10 (211.23) vs. 472.78 (182.74), *p* < 0.001; RIAP 511.16 (190.42) vs. 421.86 (156.45), *p* < 0.001]

To test whether participants improved from their first to second round of exercises, we compared the LIPL, RIPL, LIAP, and RIAP of the first round to the second round for interventional and control group separately. As we expected, in ‘Lifting and Grasping’ both, the interventional as well as the control group performed better in the second round of exercises demonstrating a training effect (Fig. 4).

**Fig. 5:**
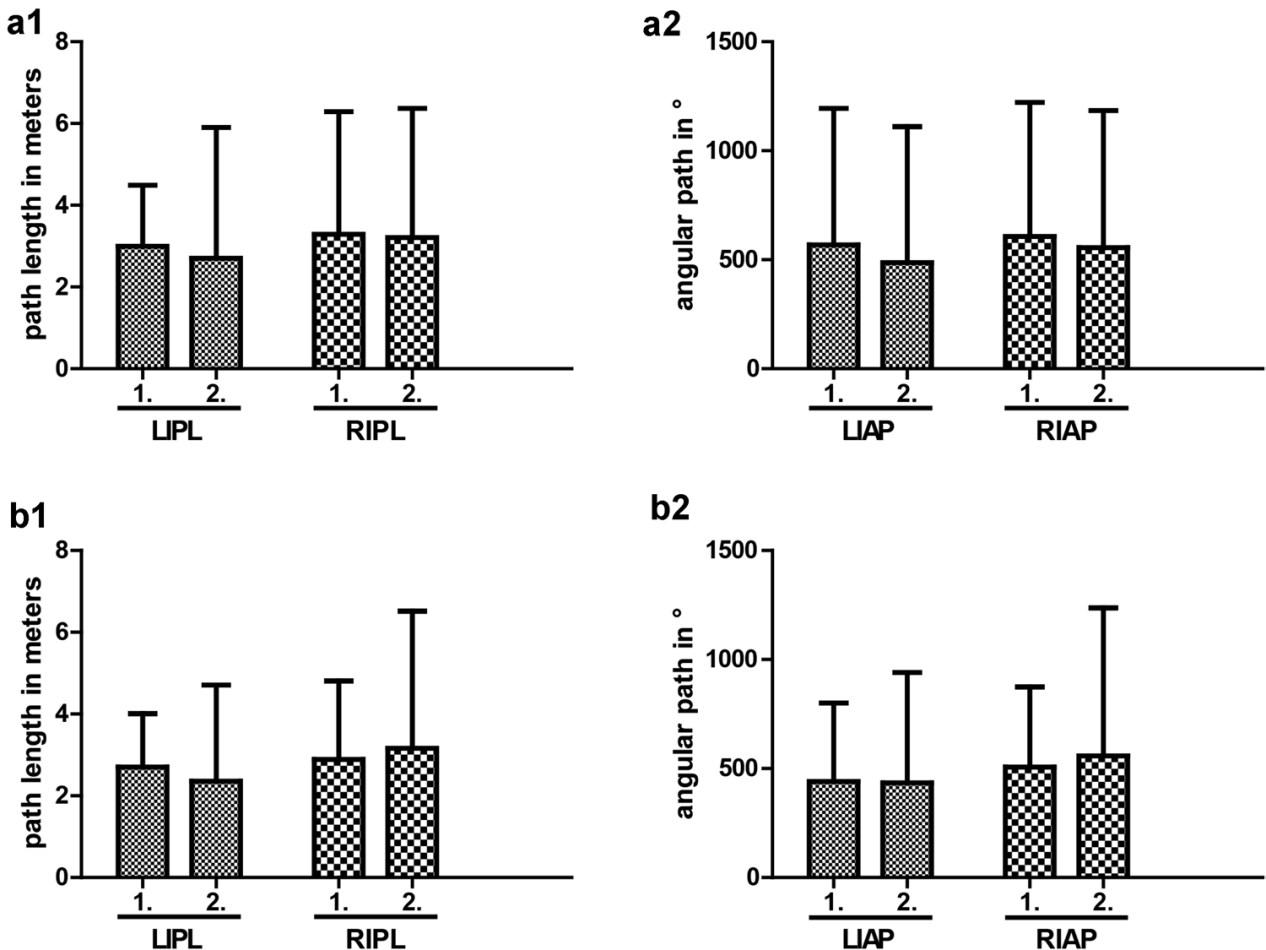
Comparison of first to second round of ‘Clip Applying’ exercise. Shorter path length and smaller angular path mean more manual dexterity. Statistical analysis was made using two-sided paired *t* testing. a—Placebo group. **a1** Path length. Subjects did not improve significantly from first to second round in path length [LIPL 3.00 (2.93) vs. 2.70 (3.20), *p* = 0.535; RIPL 3.29 (3.00) vs. 3.21 (3.16), *p* = 0.863]. **a2** Angular path. Subjects did not improve significantly from first to second round in angular path [LIAP 568.36 (625.53) vs. 487.02 (624.05), *p* = 0.391; RIAP 606.46 (615.30) vs. 555.53 (629.24), *p* = 0.577]. b—Interventional group. **b1** Path length. Subjects did not improve significantly from first to second round in path length [LIPL 2.31 (1.63) vs. 2.36 (2.35), *p* = 0.865; RIPL 2.89 (1.92) vs. 3.16 (3.36), *p* = 0.560]. **b2** Angular path. Subjects did not improve significantly from first to second round in angular path [LIAP 440.46 (360.70) vs. 433.83 (506.64), *p* = 0.929; RIAP 507.41 (366.95) vs. 558.65 (677.91), *p* = 0.577]

In ‘Clip Applying’, however, we could not observe any improvement. This might be due to ‘Clip Applying’ being a more difficile exercise which is why it might require more training to measure a difference in hand movement economy (Fig. 5).

## Discussion

The influence of caffeine intake on operative skills has been the subject of some studies. However, these were mainly done in ophthalmology [15–19], or with very small participant numbers between 5 and 22 participants [8, 15–18], or they were restricted to novice fellows or students [8, 18, 19].

The problems of internal and external validity in the current literature are why we chose to devise this study. In order to ensure a sufficiently large sample size, we conducted the study during the largest congress of surgery in Germany (annual congress of the German Society of Surgery). This also acted to ensure that experienced surgeons participated in our study. As most studies only recruited very small numbers of participants, we decided to use a set-up with only two exercises so that we could motivate a large cohort of subjects. Taken together we tested for a very long time, i.e., in total about 8 h for one exercise.

We did not measure surgical skills in real life during operations but with simulators. These guarantee patient safety and provide a possibility to measure surgical skills objectively and improve surgical skills [20, 21]. The simulator used for this study has been validated in several studies for content validity [22], concurrent validity [23], construct validity [24–26], and face validity [24]. The chosen parameters (LIPL, RIPL, LIAP, RIAP) are raw data and therefore are not further processed or interpreted by the used devices which might affect the results. Furthermore, they are validated variables for measuring manual dexterity and thereby fine motor skills [27, 28].

Our study design is a randomized placebo-controlled double-blinded trial. The first round of exercises was conducted to record a base level of performance. The following simple randomization succeeded in splitting the study group into two study groups which were equal except for caffeine consumption. Confounding factors such as age, smoking, laparoscopic experience, surgical experience, or participants’ opinion as to whether caffeine influenced their motoric skills were controlled. Additionally, we calculated the difference between the first and second round of each participant’s single hand parameters to eliminate any base level differences between the participants, thus ensuring the results were not influenced by different previous laparoscopic experience. By comparing the first and second round of exercises in each arm separately, it was clearly revealed that the simulator had a training effect at least for the easier ‘Lifting and Grasping’ exercise. In the task ‘Clip Applying’ no improvement was seen which is consistent with other studies [28] and might be due to ‘Clip Applying’ being a more complex exercise. The training effect is in accordance with previous studies [20]. Calatayud et al. showed that surgeons who do a warm up training at the simulator directly before an operation perform better during laparoscopic gall bladder resection [21].

Ingesting caffeine through drinking coffee is closest to doctors’ life reality [7] which is why we decided against caffeine tablets and for coffee as the intervention. Caffeinated filter coffee contains 0.7–1.1 mg caffeine per ml [30]. Thus, 340 ml of coffee contain at least about 230 mg caffeine [2]. Although coffee’s most obvious stimulatory component is caffeine, we cannot completely exclude that other substances had an effect on the participants additionally. By using decaffeinated coffee as a control we could ensure that caffeine was the only difference between the two study arms and any observed difference would have been based on caffeine. However, we cannot clarify if other components which are also part of decaffeinated coffee, led to a deterioration of skills in both groups compared to no coffee consumption at all. In a study, decaffeinated coffee led to increased bowel peristalsis [31]. Thus, components other than caffeine might also affect motor skills.

We decided to wait for half an hour from beginning of coffee consumption to the start of the second round of exercises as it was shown that caffeine concentration in serum was highest about 30 min after oral intake of coffee with a bioavailability of essentially 100% [3]. Additionally, doctors will likely be operating about 30 min after a coffee break in real life.

In some previous studies it is not mentioned if participants had abstained from coffee before the study [16, 18]. We included subjects who had drunk coffee before which is consistent with another study [19]. The rationale was that we wanted to ensure a normal setting as most surgeons will have their morning coffee some time before surgery and will additionally drink coffee directly before an operation or between two operations. Therefore, we wanted to investigate if a coffee break affects a surgeon’s skills regardless of their previous coffee consumption. An effect on the results was circumvented by including these participants in both arms of the study equally. Furthermore, coffee abstention might even have a greater effect on hand movement of those participants who are accustomed to a certain amount of coffee.

We decided to calculate a total effect score by combining all single hand parameters differences. By calculating the difference between the first and second round of each participant’s single hand parameters we eliminated any base level differences between the participants and thereby any confounders such as experience level or previous coffee consumption.

We could demonstrate non-inferiority between the interventional and placebo arm showing that additional caffeinated coffee consumption has no impact on laparoscopic skills. This shows that a coffee break with caffeinated coffee does not influence surgeons’ fine motor skills for laparoscopic procedures.

We only tested for laparoscopic surgery as measuring tremor in open surgery objectively is challenging. Additionally, the use of trocars might diminish the tremor effect. Therefore, our results cannot be transferred to open surgery during which caffeine might either have a bigger effect on tremor or, on the contrary, during which the tremor caused by caffeine might be irrelevant due to a greater base level of tremor which might overlay any tremor caused by caffeine.

In a subgroup analysis including those 25 participants who had abstained from caffeine for at least 8 h we found no significant difference. Although this was not our primary outcome, this result suggests that caffeine consumption has no influence on laparoscopic skills of caffeine-naïve persons.

In another subgroup analysis including those 23 participants with a very high laparoscopic experience with more than 100 laparoscopies performed we found no significant difference between the two groups. The mean age of this subgroup was substantially higher than that of the total group (42 vs. 33 years) showing that this group probably was more experienced. Though statistically not significant, there was a small difference between control and interventional group for the ‘Lifting and Grasping’ task showing a slightly higher effect score in the caffeinated group.

This might imply that caffeine only leads to a very small difference in fine motor skills which is only detectable in a simple straightforward task like ‘Lifting and Grasping’ in experienced surgeons during which they have very steady hands. In less experienced surgeons an effect by caffeine might not even be detectable as the base level of tremor is too high. Furthermore, as soon as the task gets more complex (there was no difference to be seen in ‘Clip Applying’), the effect by caffeine might be superimposed by the general unsteadiness of hands also in experienced surgeons. However, this is highly speculative and would only show that tremor caused by caffeine is only minor and clinically irrelevant. This could be explored in future by a larger study with experienced surgeons.

Consistent with our study, many previous studies found that caffeine does not lead to a change in tremor [15, 16]. Additionally, we did not find a correlation between previous coffee consumption and performance. Still, it might make a difference whether it is the first coffee in the morning after a longer abstinence overnight which we did not set as an inclusion criterion. Furthermore, the daytime when coffee is consumed might make a difference which we did not control. This is something which should be explored in future studies.

Complete caffeine abstinence might lead to an improvement according to some studies [18, 19]; however, such a proposal is not close to reality as many surgeons consume caffeine regularly. In a large study which surveyed 951 surgeons working in hospitals, lifetime, past-year, and past-month prevalence for caffeine drinks were about two thirds [7].

All in all, we verified that additional caffeinated coffee intake, e.g., during a coffee break between laparoscopic surgical procedures, does not lead to deterioration of fine motor skills’ performance.

## Supplementary Information

Below is the link to the electronic supplementary material.

Supplementary file1 (PDF 110 KB)
